# Musical Ratios in Sounds from the Human Cochlea

**DOI:** 10.1371/journal.pone.0037988

**Published:** 2012-05-24

**Authors:** Katarzyna J. Blinowska, Konrad Kwaskiewicz, W. Wiktor Jedrzejczak, Henryk Skarzynski

**Affiliations:** 1 Department of Biomedical Physics, Faculty of Physics, University of Warsaw, Warsaw, Poland; 2 Institute of Physiology and Pathology of Hearing, Warsaw, Poland; 3 World Hearing Center, Kajetany, Poland; Baycrest Hospital, Canada

## Abstract

The physiological roots of music perception are a matter of long-lasting debate. Recently light on this problem has been shed by the study of otoacoustic emissions (OAEs), which are weak sounds generated by the inner ear following acoustic stimulation and, sometimes, even spontaneously. In the present study, a high-resolution time–frequency method called matching pursuit was applied to the OAEs recorded from the ears of 45 normal volunteers so that the component frequencies, amplitudes, latencies, and time-spans could be accurately determined. The method allowed us to find that, for each ear, the OAEs consisted of characteristic frequency patterns that we call resonant modes. Here we demonstrate that, on average, the frequency ratios of the resonant modes from all the cochleas studied possessed small integer ratios. The ratios are the same as those found by Pythagoras as being most musically pleasant and which form the basis of the Just tuning system. The statistical significance of the results was verified against a random distribution of ratios. As an explanatory model, there are attractive features in a recent theory that represents the cochlea as a surface acoustic wave resonator; in this situation the spacing between the rows of hearing receptors can create resonant cavities of defined lengths. By adjusting the geometry and the lengths of the resonant cavities, it is possible to generate the preferred frequency ratios we have found here. We conclude that musical perception might be related to specific geometrical and physiological properties of the cochlea.

## Introduction

Where does our preference for certain musical intervals comes from? Why should certain frequency ratios sound musically pleasant? The issue is still unresolved [1]–[3] but Pythagoras thought the answer lay in number and geometry. Studying the vibrations of strings he found that pleasant tones are associated with small integer relationships between the string lengths. After studying otoacoustic emissions from human ears we find that Pythagoras's perspective may shed light on the physiological roots of musical perception.

The subject of consonance and dissonance is vast [1]–[3], but at base it is connected with certain combinations of particular intervals (frequency ratios) used when playing music. A rule for generating a full set of ratios is called a tuning system, and the most common western systems are equal temperament, Pythagorean, and Just. In practical terms, each does the job, but a theoretical question is why there could be more than one and which is more “correct”? The findings here lend support to the Just system, although the issue quickly becomes complex. Any tuning system relies on the human ear being sensitive to ratios of frequencies rather than to absolute differences. The ratios form musical intervals, which are steps up or down in pitch, and can be simply specified by the ratio of the frequencies involved. For example, an octave is a musical interval defined by the ratio 2∶1 regardless of the absolute starting frequency. Intervals represented by exact integer ratios are said to be Just intervals, and the temperament which keeps all intervals exact whole-number ratios is called Just intonation. The octave (2∶1), fifth (3∶2), and fourth (4∶3) are intervals which have been considered consonant throughout history by essentially all cultures. In the Just musical scale there are 12 intervals or ratios inside the octave – 16∶15, 9∶8, 6∶5, 5∶4, 4∶3, 45∶32, 3∶2, 8∶5, 5∶3, 16∶9, 15∶8, and 2∶1 (Table 1). Here we will show that the Just musical scale appears in the frequency ratios of the resonant modes of otoacoustic emissions from human ears, giving the Just scale a strong naturalistic basis.

**Table 1:** Experimentally found ratios of resonant frequencies in otoacoustic emissions compared with musical ratios of the Just scale.

Exp. ratio	Theor. ratio	Δ	Just interval	Diatonic name
1.067	1.067	0.0	16∶15	Minor second
1.125	1.125	0.0	9∶8	Major second
1.185	1.200	0.015	6∶5	Minor third
1.275	1.250	0.025	5∶4	Major third
1.374	1.333	0.041	4∶3	Perfect fourth
1.394	1.406	0.012	45∶32	Augmented fourth
1.49	1.500	0.01	3∶2	Perfect fifth
1.584*	1.600	0.016	8∶5	Minor sixth
1.661	1.667	0.006	5∶3	Major sixth
1.783	1.778	0.005	16∶9	Minor seventh
	1.875		15∶8	Major seventh
2.02	2.000	0.02	2∶1	Perfect octave

Δ difference between experimental and theoretical ratio.

* not significant.

Otoacoustic emissions (OAEs) are weak acoustic signals of cochlear origin that can be measured with a sensitive microphone in the ear canal [4]–[6]. They occur in response to acoustical stimulation or can even appear spontaneously. OAE generation involves cellular receptors in the cochlea, although the mechanism is unclear [4]. It is thought that, in response to a stimulus, the outer hair cells (perhaps with some feedback from the nervous system) cause oscillations that set in motion the basilar membrane, its fluids, ossicular chain, the ear drum, and finally the air in the ear canal. Otoacoustic emissions can be evoked by a broadband stimulus (a click) or by brief tones. In both cases they are classified as transiently evoked otoacoustic emissions (TEOAEs) and are usually averaged in response to repetitive stimulation at about 50 times a second. Since the signals are very faint, typically several hundred repetitions are used. Even without stimulation, spontaneous otoacoustic emissions (SOAEs) can arise and these are characterized by a stable amplitude and narrow bandwidth. The most common method of recording SOAEs is by synchronizing them with a broadband stimulus and averaging them in a 20 to 80 ms window following the stimulus, and such signals are called synchronized spontaneous otoacoustic emissions (SSOAE).

It is well known that in response to a broad-band click, the spectra of TEOAEs show characteristic narrow peaks [5], [6] which recur at the same frequencies in each individual, but which, like a fingerprint, differ between subjects. By using an advanced method of time–frequency analysis, it has been found that specific components of TEOAEs – characterized in terms of frequencies and latencies – can be excited by tone-bursts differing by as much as an octave [7], [8]. For any given ear, the same components – which we call “resonant modes” – appear in response to both tone-bursts and broadband stimuli [7], [8]. They can be considered a signature of each ear [9]. Investigating a large data set of SOAEs, Braun [10] found that the ratios between frequencies in an ear showed a preference for the small integers 5∶4, 6∶5, 4∶3, and 16∶15. Similarly, in earlier investigations, evidence has been found of the presence of small-integer ratios: the ratios 3∶2, 4∶3 and 2∶1 appeared between frequencies of resonant modes in TEOAEs evoked by both tone-bursts and broadband stimuli [8], [11].

In this paper, using a different set of subjects and applying an improved methodology that uses a set of asymmetric waveforms in the decomposition stage, a systematic study of the ratios between the frequencies of SSOAEs is presented. Their narrow bandwidths facilitate more precise frequency estimation than is possible for short-lasting components, a property that follows directly from the time–frequency uncertainty principle [12].

## Materials and Methods

### Subjects and data acquisition

TEOAEs from 86 ears from 45 subjects (10 males, 35 females, age 22–37, 43 right and 43 left ears) were measured under low ambient noise conditions using an ILO-96 apparatus (Otodynamics Ltd, Hatfield, U.K.). All participants presented normal middle ear function, normal pure tone audiometry thresholds (≤20 dB HL for frequencies of 0.25, 0.5, 1, 2, 4, and 8 kHz), and had no retrocochlear complications. Standard click stimuli with average amplitudes 80±3 peak dB SPL, using a nonlinear averaging protocol, were used. Each analyzed signal was an average of 520 OAE responses (twice the standard, which is 260). The initial part of the response was windowed automatically (onset of the window was set at 3.6 ms), thus minimizing the influence of the stimulus artifact on the output signal. Recordings were performed in a window of 80 ms. To reduce low-frequency noise, the signals were high-pass filtered above 500 Hz. Only records with at least two long-lasting (spontaneous) components were kept for further analysis, which effectively yielded 86 records out of a possible 90 (4 subjects had detectable long-lasting components in one ear only). These components are described in detail below in the section “Selection of SSOAE components”. The study was approved by the ethics committee of the Institute of Physiology and Pathology of Hearing.

### Method

The method used here which allowed us to identify resonant modes is called adaptive approximations by matching pursuit (MP). The method is based on the adaptive decomposition of a signal into its basic waveforms characterized in terms of frequency, amplitude, latency (or time of occurrence), and time-span (duration); it can also yield an asymmetry parameter.

The MP algorithm was introduced in 1993 [13] and was first applied to physiological signal processing a year later [14]. The method is very robust in respect to noise. The addition of noise with a variance twice as large as the variance of the signal does not appreciably influence the time–frequency positions of waveforms corresponding to simulated structures; only some spurious waveforms are added [15]. Advantages of the method have been demonstrated in EEG studies where MP has found a broad range of applications: e.g., for extraction of specific structures from the signal [16] and for revealing microstructure of event-related responses [17]. Application of MP to sleep analysis has allowed a description, in the same framework, of transient and oscillatory structures of the signal [18] and has made possible the construction of a fully automatic sleep-staging system [19].

The MP method has superior time–frequency resolution compared to other methods like windowed Fourier transform, Wigner-Ville transform, and wavelets, capabilities which have already been demonstrated in the context of OAEs in [7], [8]. MP is a powerful method that has proved useful in explaining phenomena such as TEOAE suppression [20] and the longer OAE latencies of preterm newborns [21].

### Matching Pursuit algorithm

The MP method relies on iterative decomposition of a signal into waveforms from a very broad and redundant dictionary of functions. Since it is an NP-hard problem (computationally intractable), the iterative sub-optimal procedure is applied. In the first step of the iterative procedure the vector 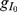 is chosen which gives the largest product with the signal *f(t)*:
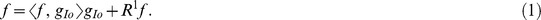
Then the residual vector *R^1^* obtained after approximating *f* in the direction 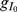 is decomposed in a similar way. The iterative procedure is repeated on the ensuing residues:
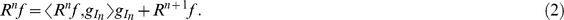
In this way the signal *f* is decomposed into a sum of time–frequency waveforms,
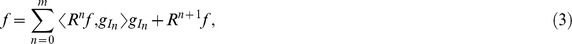
chosen to match optimally the signal's residues.

The point at which the iterations are stopped, or equivalently, the number of waveforms in expansion (3), can be chosen individually for each signal (based on mathematical criteria or set arbitrarily, e.g., as a percentage of the energy accounted for). It has been proven^1^ that the procedure converges to f, and that the energy of representation is conserved so that:
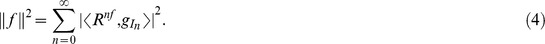



A dictionary of basic waveforms is generated by scaling, translating, and (unlike in wavelet transform) *modulating* the window function *g(t)*:
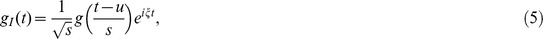
where *s*>0 is scale, ξ is frequency modulation, and *u* is translation.

In practice, for analysis of real-valued signals, the dictionaries are limited to real functions. Index *I = (ξ, s, u)* describes the set of parameters. The dictionaries of windowed Fourier transform and wavelet transform can be derived as subsets of this dictionary, defined by certain restrictions on the choice of parameters. In the case of the windowed Fourier transform, the scale *s* is constant – equal to the window length – and the parameters ξ and *u* are uniformly sampled. In the case of WT, the frequency modulation is limited by the restriction on the frequency parameter ξ = ξ_0_/*s*, where ξ_0_ = *const*.

The highest time–frequency resolution (close to the one determined by the uncertainty principle) is obtained for functions *g_I_* from the Gabor family [13], so usually this kind of function is applied. However, the best representation is obtained when basic functions match the signal's components. Since long-lasting components of OAE are asymmetric, with a fast-rising initial part and slowly decaying tail, functions of asymmetric shape were introduced [22]:
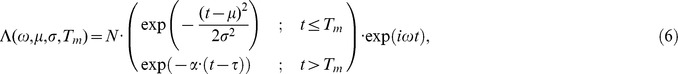
where 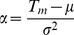 and 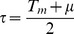, and ω is frequency, μ is latency, σ is time-span, *T_m_* is the point where the Gaussian envelope changes into an exponent, and *N* is a normalization constant. The function obtained in this way is continuous up to the first-order derivative.

Introducing asymmetric waveforms brings several advantages, and one of them is sparsity of representation. For a dictionary consisting of Gabor functions, the long-lasting components are sometimes described by more than one waveform, since the standard MP algorithm tends to split such activity into two components: one with high amplitude and short duration (related to response onset), and a second spanning through the whole length of the signal (related to the tail of the decaying activity) [23]. In the framework of an enriched dictionary containing asymmetric functions, long-lasting components are described by a single function. Another advantage of asymmetric functions is the correct estimation of the latency of long-lasting components, and elimination of “pre-echo” effects (creation of energy before the start of the actual signal) [22].

**Figure 1:**
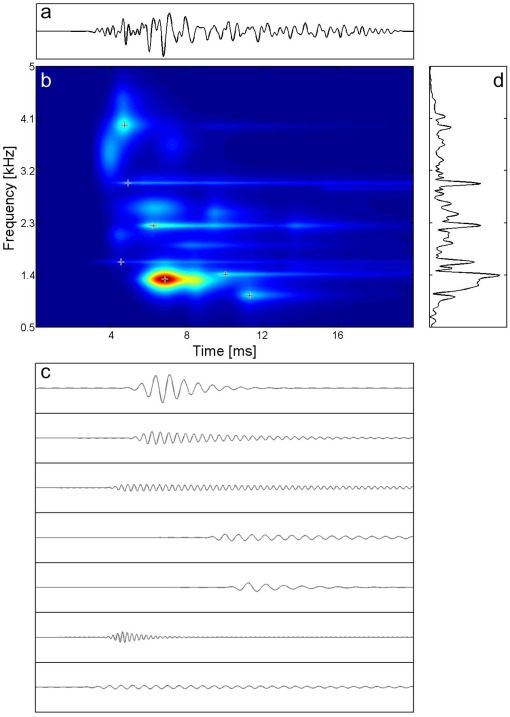
Decomposition of a TEOAE signal and its time–frequency representation. (a) The average TEOAE signal; (b) its time–frequency distribution; (c) the 7 strongest components of the TEOAE found by means of the MP method; (d) Fourier spectrum of the signal. Comparison of (b) and (d) shows that the conventional method of OAE analysis gives limited information in comparison with MP.

The set of functions from which the waveforms were fitted can be very large: in our case it consisted of 7×10^7^ possible waveforms comprised of Gabor functions (Gaussian modulated sinusoids) and also asymmetric functions. From the defined set of functions, waveforms of varying shapes were iteratively fitted onto a fine time–frequency grid. The time-step was flexible (0.6 ms on average) and the frequency step was 12.5 Hz. No particular frequency was privileged. Iteration was continued until 95% of the signal energy was accounted for. Figure 1 is an example of the MP approach and shows how it identifies basic waveforms present in the signal and represents them on a high-resolution time–frequency plot.

### Selection of SSOAE components

The MP decomposition provides not only the frequency and latency of a signal's components, but also its time-span. It has been found previously that TEOAE waveforms exhibit a bimodal distribution: there are short-lasting ones with time-spans less than 10 ms and long-lasting ones with time-spans greater than 10 ms [7], [24]. The long-lasting components are of narrow bandwidth and are known as synchronized spontaneous otoacoustic emissions (SSOAEs); they are directly related to spontaneous otoacoustic emissions (SOAEs) [25], [26]. SSOAEs are routinely measured some 20 ms after stimulus onset, when the short-lived components have disappeared. Our method makes it possible to observe SSOAEs over the whole epoch of measurement, since it provides a parametric description of their components, including their time-spans. This paper focuses on these long-lasting components.

### Calculation of the errors in frequency ratios

The error of the ratio *f*_1_/*f*_2_ was calculated as:
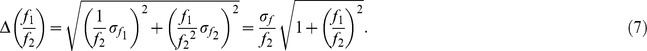



The accuracy of fitting an atom in the MP procedure was 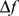 = 12.5 Hz. It was assumed that the distribution of frequencies within a single bin was uniform. Then 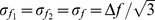 is the error in estimating the frequency components *f*_1_ and *f*_2_. Since a given ratio can come from a combination of different frequencies, its accuracy differs depending on *f*_1_ and *f*_2_. Taking into account the range of considered frequencies from 500 Hz to 4000 Hz, the maximal error is 0.044.

## Results

**Figure 2:**
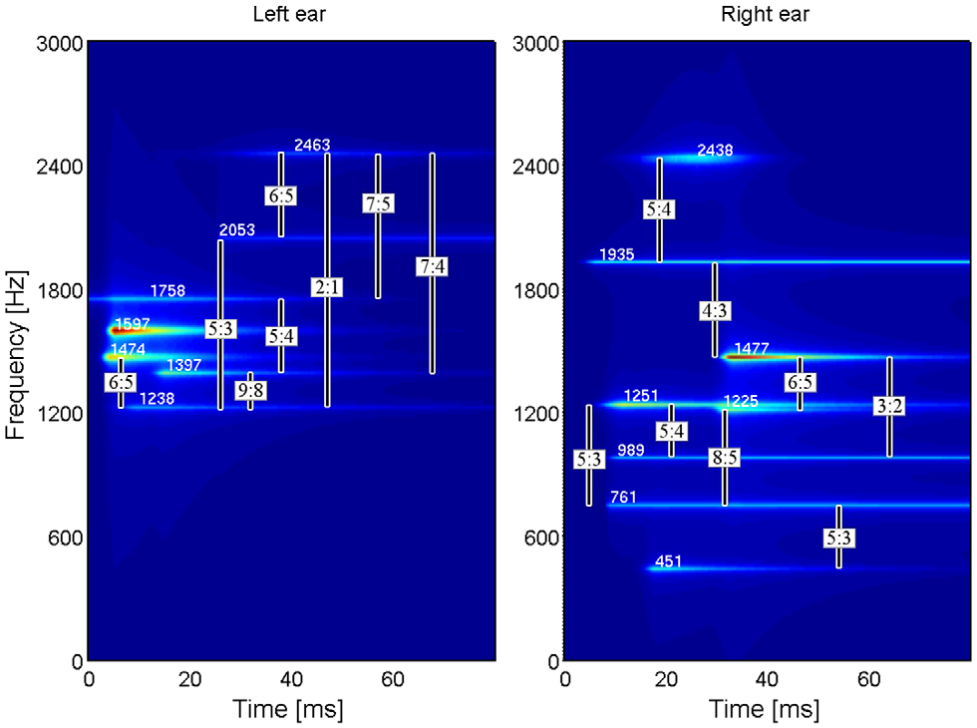
Time–frequency representation of TEOAEs for the left and right ears of one subject. The amplitudes of resonant modes are color coded. The vertical bars connect resonant modes having small-integer ratios.

The MP method was applied to 86 TEOAE records. From the decompositions long-lasting components were picked out, i.e. waveforms with time-span >10 ms (half-width). Each of the 86 records had at least 2 long-lasting waveforms (on average, 4.7 per record). An example of a time–frequency representation of a TEOAE for the left and right ear of a subject with multiple SSOAEs is shown in Fig. 2. It is apparent that different resonant modes occur in each ear, and ratios between the frequencies of some of them are marked. A particular frequency ratio may appear in one or both ears; however no prevalence of particular frequency ratios between ears was observed.

**Figure 3:**
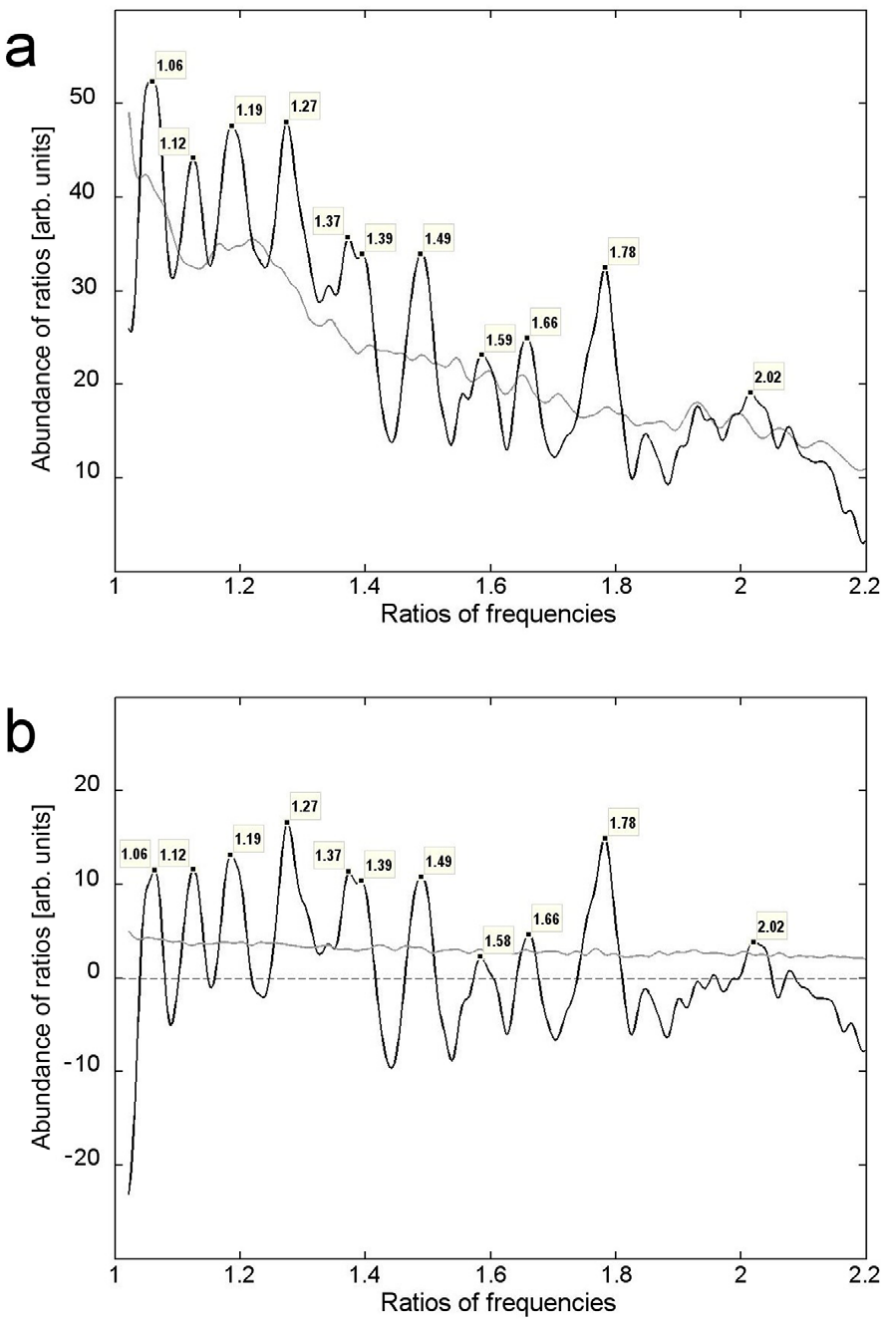
Musical-interval structure of long-lasting TEOAE components. (a) Distribution of ratios of OAE frequencies found for individual ears (black line) and distribution of ratios determined from randomly drawn OAE frequencies (gray line); (b) difference (black line) between the curves shown in *a*. The gray line shows the 75th percentile of the distribution of ratios of randomly drawn frequencies.

In order to statistically prove the occurrence of integer frequency ratios between resonant modes for each individual ear, all possible frequency ratios were calculated. Next, a histogram was constructed of all the ratios obtained from individual ears. In total 867 ratios were obtained, and the black line in Fig. 3a shows their distribution. The distribution has been smoothed using a moving window of width 0.04 and step-size 0.0008. The position of each peak is marked.

In the next step a bootstrapping technique [27] was used to evaluate the significance of the peaks. Namely, an estimation was made of what the distribution of ratios would be if they occurred randomly. To do this the frequencies found for all ears were pooled and from that pool frequencies at random were withdrawn and their ratios calculated until 867 random ratios were obtained. This procedure was repeated 1000 times to produce 1000 distributions of random ratios, which allowed us to plot a mean random distribution, shown as the gray line in Fig. 3a.

To estimate the significance of the results, the random background was subtracted from the distribution obtained from individual ears (the gray line in Fig. 3 was subtracted from the black line) and the result is shown in Fig. 3b. Significant peaks are those which deviate from the random line at the 75% percentile (gray line in Fig. 3b) of the random distribution.

Fig. 3b shows 10 clearly defined peaks which appear above the threshold of the 75th percentile. Moreover, the *x*-values of these peaks are close to small-integer ratios, reflecting those of Just intervals in the diatonic scale (see Table 1). Only one peak (1.58) corresponding to the minor sixth (8∶5) appears below the 75th percentile, and the peak at 1.875 corresponding to the major seventh (15∶8) is missing.

In Table 1 the experimentally found ratios of OAE resonant frequencies are compared with the ratios of the Just scale. The differences between the theoretical values corresponding to Just intervals and experimental results are generally small. The biggest discrepancy (1.37 compared to 1.33) appears for the perfect fourth (4∶3) and is probably connected with the closeness of peaks corresponding to the perfect fourth (4∶3) and augmented fourth (45∶32).

The determination of errors in the ratios of the peaks is difficult, since the errors depend on the frequencies from which each ratio was calculated. For example, the ratio 1.5 may come from the frequencies 3 kHz and 2 kHz or from the frequencies 0.9 kHz and 0.6 kHz. In the case of 3 kHz/2 kHz, the error will be 0.011; in the case of 0.9 kHz/0.6 kHz it will be 0.022.

It may be seen from Fig. 3 and Table 1 that almost all the ratios corresponding to the diatonic scale are present in the distribution – the correspondence is striking. It leads to the conclusion that the human cochlea appears to contain resonant elements whose tuning reflects the 12-semitone Just scale. The occurrence of ratios 16∶15, 6∶5, 5∶4, 4∶3, 3∶2, and 2∶1 in SSOAEs has been reported earlier [8], [10], [11]; however, in these cases the evidence did not always carry statistical weight. In [8], [11] the search for 3∶2, 4∶3, and 2∶1 was undertaken, and only these ratios were identified. Here, in a statistically significant way, the existence of 10 out of the 12 intervals from the Just scale was confirmed. These results are more strongly justified than the ones from [8], [11], since here an improved methodology was applied, particularly the application of asymmetric waveforms to characterize long-lasting components. A dictionary consisting only of symmetric atoms may result in splitting the long component into several waveforms, which might disturb the results [22]. The identification of practically all the main intervals from the Just scale in different experimental material further validates the results.

## Discussion

From the above evidence it may be conjectured that our perception of music is connected to the same mechanisms operating in the inner ear which produce otoacoustic emissions.

It is generally thought that the perception of musical intervals is connected with high-level cortical processing; however it has been recently found that representation of musical pitch in humans is present at a subcortical level [28], [29], namely at the level of the brainstem. It has been suggested [29] that preattentive, sensory-level processing may account for the perception of consonance. Indeed, the present findings suggest that the Pythagorean ratios may be somehow encoded in the cochlea, perhaps giving a universal naturalistic basis for music. Interestingly, a *perfect fifth* (3∶2) and a *perfect fourth* (4∶3) are present in both European and oriental music [30] and even speech [31].

Although OAEs form the basis of a widely applied test for hearing screening, the underlying mechanisms are still a matter of debate. The issue relates to two major models, the traveling wave theory [32]–[34] and the resonance theory [35]–[37]. The traveling wave may provide a rough mechanism for frequency selectivity; however the high sensitivity and fine tuning of the ear require active mechanisms derived from resonant vibrations of hair cells. The presence of an active feedback mechanism connected with the motility of outer hair cells (OHCs) has been confirmed by biomedical evidence [38], [39]. Effects due to the mechanical responses of OHCs have been modeled [40] and these studies indicate that OHCs can act as active amplifiers of membrane oscillations in the inner ear.

Anatomical studies of OHCs in the cochlea have found a regular, almost crystal-like, geometrical arrangement [41]. Most of the attempts to explain the fine structure of OAEs have assumed some unspecified random inhomogeneities or roughness, an approach that presents difficulties in predicting the precise positions of OAE spectral lines. It seems unlikely that the striking structural arrangement of the OHC pattern – rows with a well-defined orientation – is not purposeful. An intriguing theory that accounts for the specific arrangement of the OHCs has been presented by Bell [42]. The theory presents a physical model of the cochlea as a surface acoustic wave resonator in which spacing between the rows of OHCs creates resonant cavities of defined lengths, just like the spacing of interdigital electrodes controls the resonance frequency in an electronic equivalent. By examining published micrographs [41], Bell conjectured that reverberating OHCs could produce cavities of specific lengths, orientations, and frequencies [42]. The interesting aspect is that, by simple geometry, the cavity lengths (the inverse of frequency) and their ratios reflect the musical scale. Although still speculative, our findings are neatly explained by the above model, and our favored interpretation is that cochlear tuning derives from standing waves between the rows of OHCs.

Since the receptors of the inner ear – the outer hair cells – get feedback from the central nervous system, neural influences might also contribute to cochlear tuning. Whether or not this is the case, the attractive feature of the standing wave model is that it points to musical ratios arising directly from anatomically defined inter-cell lengths, a notion not far from the original plucked string idea formulated by Pythagoras. We are led to conjecture that our perception of music is connected with anatomical and mechanical properties of the inner ear, an organ which appears to operate as a very subtle, highly tuned active acoustic resonator. The Pythagorean statement connecting music with geometry might have real physical meaning, since the results presented here raise the possibility that music might have its roots within the structure of the human ear.

## References

[1] Zatorre RJ, Peretz I (2001). The Biological Foundations of Music.. Ann N Y Acad Sci.

[2] Anon (2008). Science & Music.. http://www.nature.com/nature/focus/scienceandmusic/.

[3] Shapira Lots I, Stone L (2008). Perception of musical consonance and dissonance: an outcome of neural synchronization.. J Roy Soc Interface.

[4] Kemp DT, Manley GA, Fay RR, Popper AN (2008). Otoacoustic emissions: concepts and origins.. Active Processes and Otoacoustic Emissions.

[5] Elberling C, Parbo NJ, Johnsen NJ, Bagi P (1985). Evoked acoustic emissions: clinical application.. Acta Oto-Laryngol.

[6] Probst R, Lonsbury-Martin BL, Martin GK (1991). A review of otoacoustic emissions.. J Acoust Soc Am.

[7] Jedrzejczak WW, Blinowska KJ, Konopka W, Grzanka A, Durka PJ (2004). Identification of otoacoustic emission components by means of adaptive approximations.. J Acoust Soc Am.

[8] Blinowska KJ, Jędrzejczak WW, Konopka W (2007). Resonant modes of otoacoustic emissions.. Physiol Meas.

[9] Jedrzejczak WW, Blinowska KJ, Konopka W (2006). Resonant modes in transiently evoked otoacoustic emissions and asymmetries between left and right ear.. J Acoust Soc Am.

[10] Braun M (1997). Frequency spacing of multiple spontaneous otoacoustic emissions shows relation to critical bands: a large-scale cumulative study.. Hear Res.

[11] Blinowska KJ, Jedrzejczak WW, Konopka W (2005). Resonant modes and musical ratios in otoacoustic emissions.. Biol Cybern.

[12] Pinsky MA (2002).

[13] Mallat SG, Zhang Z (1993). Matching pursuit with time–frequency dictionaries.. IEEE Trans Sign Process.

[14] Blinowska KJ, Durka PJ, Dagli CH, Fernandez BR (1994). The application of wavelet transform and matching pursuit to the time-varying EEG signals.. Intelligent Engineering Systems through Artificial Neural Networks. Vol 4.

[15] Blinowska KJ, Durka PJ (2001). Unbiased high resolution method of EEG analysis in time–frequency space.. Acta Neurobiologiae Experimentalis.

[16] Zygierewicz J, Blinowska KJ, Durka PJ, Szelenberger W, Niemcewicz S (1999). High resolution study of sleep spindles.. Clin Neurophys.

[17] Durka PJ, Ircha D, Neuper Ch, Pfurtscheller G (2001). Time–frequency microstructure of event-related EEG desynchronization (ERD) and synchronization (ERS).. Med Biol Eng Comput.

[18] Malinowska U, Durka PJ, Blinowska KJ, Szelenberger W, Wakarow A (2006). Micro- and macrostructure of sleep EEG.. IEEE Eng Med Biol Mag.

[19] Malinowska U, Klekowicz H, Wakarow A, Niemcewicz S, Durka PJ (2009). Fully parametric sleep staging compatible with the classic criteria.. Neuroimage.

[20] Jedrzejczak WW, Smurzynski J, Blinowska KJ (2008). Origin of suppression of otoacoustic emissions evoked by two-tone bursts.. Hear Res.

[21] Jedrzejczak WW, Hatzopoulos S, Martini A, Blinowska KJ (2007). Otoacoustic emissions latency difference between full-term and preterm neonates.. Hear Res.

[22] Jedrzejczak WW, Kwaskiewicz K, Blinowska KJ, Kochanek K, Skarzynski H (2009). Use of the matching pursuit algorithm with a dictionary of asymmetric waveforms in the analysis of transient evoked otoacoustic emissions.. J Acoust Soc Am.

[23] Notaro G, Al-Maamury AM, Moleti A, Sisto R (2007). Wavelet and matching pursuit estimates of the transient-evoked otoacoustic emission latency.. J Acoust Soc Am.

[24] Jedrzejczak WW, Blinowska KJ, Kochanek K, Skarzynski H (2008). Synchronized spontaneous otoacoustic emissions analyzed in a time-frequency domain.. J Acoust Soc Am.

[25] Wable J, Collet L (1994). Can synchronized otoacoustic emissions really be attributed to SOAEs?. Hear Res.

[26] Prieve BA, Falter SR (1995). COAEs and SSOAEs in adults with increased age.. Ear Hear.

[27] Chernick MR (2007). Bootstrap Methods: A guide for researchers and practitioners, 2nd edition.

[28] Lee K, Skoe E, Kraus N, Ashley R (2009). Selective subcortical enhancement of musical intervals in musicians.. J Neurosci.

[29] Bidelman G, Krishnan A (2009). Neural correlates of consonance, dissonance, and the hierarchy of musical pitch in the human brainstem.. J Neurosci.

[30] Carterette EC, Kendall RA, Deutsch D (1999). Comparative music perception and cognition.. The Psychology of Music.

[31] Schwartz DA, Howe CQ, Purves D (2003). The statistical structure of human speech sounds predicts musical universals.. J Neurosci.

[32] Zweig G, Shera CA (1995). The origin of periodicity in the spectrum of evoked otoacoustic emissions.. J Acoust Soc Am.

[33] Shera CA, Guinan JJ (1999). Evoked otoacoustic emissions arise by two fundamentally different mechanisms: A taxonomy of mammalian OAEs.. J Acoust Soc Am.

[34] Talmadge CL, Tubis A, Long GR, Tong C (2000). Modeling the combined effects of basilar membrane nonlinearity and roughness on stimulus frequency otoacoustic emission fine structure.. J Acoust Soc Am.

[35] Sisto R, Moleti A (1999). Modelling otoacoustic emissions by active nonlinear oscillators.. J Acoust Soc Am.

[36] Nobili R, Vetesnik A, Turicchia L, Mammano F (2003). Otoacoustic emissions from residual oscillations of the cochlear basilar membrane in a human ear model.. J Assoc Res Otolaryngol.

[37] Bell A, Fletcher NH (2004). The cochlear amplifier as a standing wave: Squirting waves between rows of outer hair cells?. J Acoust Soc Am.

[38] Ashmore JF (1987). A fast motile response in guinea-pig outer hair cells: the cellular basis of the cochlear amplifier.. J Physiol.

[39] Brownell WE (1990). Outer hair cell electromotility and otoacoustic emissions.. Ear Hear.

[40] Neely ST, Kim DO (1990). A model for active elements in cochlear biomechanics.. J Acoust Soc Am.

[41] Lonsbury-Martin BL, Martin GK, Probst R, Coats AC (1998). Spontaneous otoacoustic emissions in a nonhuman primate. II. Cochlear anatomy.. Hear Res.

[42] Bell A, Stevens C, Burnham D, McPherson G, Schubert E, Renwick J (2002). Musical ratios in geometrical spacing of outer hair cells in the cochlea: strings of an underwater piano?. Proc Int Conference on Music Perception and Cognition.

